# Conditional Cash Transfers for Maternal Health Interventions: Factors Influencing Uptake in North-Central Nigeria

**DOI:** 10.15171/ijhpm.2018.56

**Published:** 2018-06-25

**Authors:** Fatima Baba-Ari, Ejemai Amaize Eboreime, Mazeda Hossain

**Affiliations:** ^1^Department of Primary Healthcare Systems Development, National Primary Health Care Development Agency, Abuja, Nigeria.; ^2^Presidential Initiative for the North East (PINE), Abuja, Nigeria.; ^3^Department of Planning, Research and Statistics, National Primary Health Care Development Agency, Abuja, Nigeria.; ^4^Centre for Health Policy, Wits School of Public Health, University of the Witwatersrand, Johannesburg, South Africa.; ^5^Department of Global Health and Development, London School of Hygiene and Tropical Medicine, London, UK.

**Keywords:** Conditional Cash Transfer, Health Financing, Maternal Health, Nigeria

## Abstract

**Background:** Nigeria accounts for a significant proportion of global maternal mortality figures with little progress made in curbing poor health indices. In a bid to reverse this trend, the Government of Nigeria initiated a conditional cash transfer (CCT) programme to encourage pregnant women utilize services at designated health facilities. This study aims to understand experiences of women who register for CCT services and explore reasons behind non-uptake of those women who do not register.

**Methods:** We conducted this study in a rural community in North Central Nigeria. Having identified programme beneficiaries by randomly sampling contact details obtained from the programme database, using snowball sampling method we sourced non-beneficiaries list based on recommendations from beneficiaries and other community members. Thereafter we undertook semi-structured interviews on both beneficiaries and non-beneficiaries and analysed data obtained thematically.

**Results:** Our findings revealed that, while beneficiaries of the programme were influenced by the cash transfers, cash may not be sufficient incentive for uptake by non-beneficiaries of CCT in Nigeria. Factors such as community and spousal influence, availability of free drugs, proximity to health facility are critical factors that affect uptake in our study context. On the other hand, poor programme administration, mistrust for government initiatives as well as poor quality of services could significantly constrain service utilization despite cash transfers.

**Conclusion:** Considering that a number of barriers to uptake of the CCT programme are similar to barriers to maternal health services, it is essential that maternal health services are available, accessible and of acceptable quality to target recipients for CCT programmes to reach their full implementation potential.

## Background

Available evidence indicates supply-side interventions alone are not sufficient to adequately increase uptake of healthcare services if demand side limitations, such as the population’s poverty level and user fees in health facilities, are not taken into consideration.^1^ Consequently, governments are increasingly using financial incentives to improve utilization of health services across the developing world.^2,3^ Conditional cash transfer (CCT) programmes in healthcare operate on the premise of providing financial incentives to its users to promote health-seeking behaviour. The beneficiaries of CCTs are targeted beneficiaries who meet specified conditions thus the CCT programmes in health are usually designed to target specific populations (eg, pregnant women) and expected to have positive impact on their health. Thus, CCTs work by concurrently reducing financial barriers and boosting behavioural change: specifically health-seeking behaviour in this study.

De Janvry and Sadoulet opine that the CCT approach is considerably more effective than unconditional cash transfers in altering behaviour once it is established that imposing a condition on behaviour is acceptable, such as in the case of information asymmetry, which is common in healthcare, more so in low- and middle-income country (LMIC) contexts. They further opine that the effectiveness of CCTs is also dependent on some other pre-considerations such as ensuring availability of supply-side of services. In addition, cost of altering behaviour must be much lower through a price than an income effect, thus the opportunity cost of utilizing services should be a key consideration.^4^ Despite these, Fenwick posits that institutionalizing effective national social safety nets such as CCTs depends largely on the broader contextual implementation environment than on the technical design and merits of the interventions.^5,6^

CCT programmes have gained increasing popularity and have been recently implemented in several developing countries such as Kenya, Malawi, Cambodia, South Africa, and impact evaluations from these countries show increased health service utilization.^7^ Studies from various countries where CCT has been implemented, particularly to improve utilization of maternal health services, have shown varying degrees of effectiveness as it relates to their various sociocultural contexts.^2,3,8-11^ Theoretically, conditional cash incentives in healthcare are expected to subsidise costs of healthcare services,^12^ thus leading to a rise in demand in the provided health services. This does not always happen as expected and the literature is sparse on understanding why some CCT programmes show better results than others.^13^ Some studies identify low programme awareness as impeding demand for services.^14^ In rural parts of India, this has been attributed to the literacy status of women and their spouses.^15^ Transportation difficulties and previous maternal experiences of successful non-institutional deliveries have also been associated with the non-use of CCT interventions.^16^ In some contexts, poor infrastructure and poor quality of care, compounded by programme constraints such as challenges in accessing cash incentives (including perceived corrupt practices in disbursements), affected utilization of CCT programmes.^17^ Little research has, however, been carried out qualitatively to explore perceptions of the community members and stakeholders to understand factors and mechanisms influencing effective implementation of CCT programmes, particularly in sub-Saharan Africa.^18-20^

Nigeria, a country in which one third of its women (mostly rural dwellers) receive no antenatal service,^21^ introduced a pilot CCT programme in selected rural primary health facilities nationwide, in a bid to improve utilization of maternal healthcare services as part of the *Subsidy Reinvestment and Empowerment Programme: Maternal and Child Health Component* (SURE-P MCH). Launched on January 1, 2012, SURE-P MCH combines supply-side measures, such as the provision of qualified midwives to previously understaffed facilities, with demand-side measures including behaviour change communication and a CCT scheme targeting pregnant women. The SURE-P MCH was designed to focus on the provision of social protection/safety net initiatives affecting maternal and child health. Nigeria’s CCT programme provides monetary incentives to encourage pregnant women to go through the full continuum of MNCH services from antenatal care (ANC) to skilled birth delivery and postnatal care for the mothers and newborns (Table 1). The programme utilised geographic targeting, by registering pregnant women in receiving healthcare services from SURE-P designated primary healthcare (PHC) facilities in rural areas and not based on their socio-economic status. This avoided high administrative costs usually perceived in cash transfer programmes that target by socio-economic characteristics, as this information is usually not available at the household level in Nigeria. The CCT, through provision of this incentive, sought to increase pregnant women’ welfare by providing them with cash support to reduce the impact of economic barriers to access to health services (eg, transportation costs to the PHC). The total incentive amount is N 5000 (approximately US$30) per woman, conditioned upon fulfilment of outlined co-responsibilities. This rate was attained by appraising the regular cost of out-of-pocket expenditures and transportation expenses to health facilities by pregnant women, based on community surveys and focus group discussions conducted during the pre-pilot planning of the programme.^22^ An additional incentive is the provision of free (basic) maternity care to women in the designated PHCs to further improve demand for these health services.^18^

**Table 1:** CCT Incentive and Eligibility Criteria

**Cash Transfer Per Beneficiary**		**Eligibility Criteria**
**Naira**	**Dollars** ^a^	
N1000	$5	Registration for CCT programme and first ANC visit at designated PHC
N1000	$5	Second, third and fourth ANC visits at PHC
N2000	$10	Skilled Birth Delivery
N1000	$5	Postnatal care/Immunization/Family planning advice

Abbreviations: CCT, conditional cash transfer; PHC, primary healthcare; ANC, antenatal care. ^a^ Conversion rate (1/1/2014): $1= N199.

The CCT programme was piloted in nine states selected to provide representation from each of the six geopolitical zones. A cluster of four PHCs and one general hospital in each state was selected with sufficient existing infrastructure and human resources for health to be able to handle the basic requirements of the pilot. There are four midwives at each PHC facility employed by the SURE-P MCH programme to provide maternal health services for the target community. Community health extension workers are also employed at the PHC but mainly conduct outreach services within the communities.

Evidence showed some success with the Nigerian pilot CCT programme early in its implementation in terms of increased monthly number of women attending four or more ANC visits and increased monthly number of women receiving two or more Tetanus toxoid doses during pregnancy. Other outcomes such as skilled birth attendance were unaffected.^18^

Our study, conducted in a rural community in North Central Nigeria, sheds light on factors affecting the uptake of the CCT programme in Nigeria by providing the perspectives of target beneficiaries on what works, thus filling the knowledge gap in this regard.

## Methods

This qualitative study explored experiences of *beneficiaries* and perceptions of women who were eligible and aware but did not participate (*non-beneficiaries*) in CCT programme in Nigeria. A qualitative approach to data collection was used since the aim of the study was to construe the individual experiences and understandings of the study participants regarding the CCT programme. Use of qualitative methods offers a different viewpoint and supplements existing research.^19^ This study is approached largely from descriptive phenomenological perspective.^23^ Thus, this research describes participants’ subjective perceptions and experiences with the programme to understand reasons behind decisions to register or not register. Primary data collection was conducted through semi-structured interviews with these stakeholders within their communities using open-ended questions (See Supplementary file 1). Responses were audio recorded or field notes taken in some cases. Semi-structured interview method of data collection was employed because of time constraints and because it ensured flexibility as well as the maximum generation of data for the study since there was limited published information on the study topic.^24^ The interviews were all conducted in English except for two participants (P4 and P5) who only understood Hausa which was interpreted by the interviewer who is a native Hausa speaker. The interviews were all conducted by the lead project researcher with assistance from a SURE-P MCH programme officer.

### Sampling

The study was carried out in Byazhin PHC located in a rural community in Nigeria’s Federal Capital Territory. This site was selected purposively for the sociocultural significance of its indigenous population (the Gbagyi tribe) in North Central region of Nigeria. The Gbagyi people are spread across 6 of the 7 states in the region and are the predominant tribe in Nigeria’s capital.^25^ Twelve interviews were conducted using an audio recorder and field notes to maximise reliability. The Byazhin community was conveniently sampled due to time constraints of the project and security concerns in the country. Beneficiaries were randomly selected from the SURE-P MCH official database of beneficiaries and were contacted by phone initially and details of the study explained for consent to participate. This method of selection was purposeful to determine appropriate participants who can best inform and enhance understanding of the research question. Non-beneficiaries were sampled from identified beneficiaries and health workers at the Byazhin PHC facility using snowballing technique. All participants were female and aware of the CCT program being implemented at the PHC.

A total of 21 individuals were contacted to participate in the study of which 18 were eligible to partake in the study and 12 agreed to participate. The demographic details of these non-respondents did not differ widely from the respondents. Two of the non-respondents who were non-beneficiaries of the scheme were Muslims while two were not aware of the programme when they were pregnant. Twelve respondents are considered an adequate sample size in qualitative research since the aim of sampling is for producing maximum depth of data that answers the research question and not necessarily to represent the population.^24^ Further, this number was deemed adequate after achieving data saturation with the identified participants. Participants were divided into two categories: those that received the CCT incentive (beneficiaries) and those that did not receive the incentive but were aware of the scheme (non-beneficiaries). Some non-beneficiaries identified by snowball method were not included in the study because they were unaware of the CCT scheme. Participants are identified by codes P1-P12, with P1-P6 indicating beneficiaries and P7-P12 indicating non-beneficiaries (Table 2).

**Table 2:** Characteristics of Study Participants

**Participant**	**Age**	**Marital Status**	**No. of Children**	**Religion**	**Education Level**	**Occupation**	**Distance to Health Facility**
**CCT Beneficiaries**							
P1	27	Married	5	Christianity	Primary	Housewife	Less than 5 km
P2	30	Married	5	Islam	Secondary	Local trader	More than 5 km
P3	40	Married	7	Christianity	Primary drop-out	Housewife	More than 5 km
P4	33	Married	4	Christianity	Nil	Housewife	Less than 5 km
P5	28	Married	5	Islam	Nil	Housewife	More than 5 km
P6	30	Married	6	Christianity	Secondary	Hairdresser	Less than 5 km
**Non-CCT Beneficiaries**							
P7	32	Married	5	Christian	Primary	Farmer	Less than 5 km
P8	26	Married	3	Christian	Primary	Market seller	More than 5 km
P9	25	Married	5	Islam	Secondary	Housewife	More than 5 km
P10	34	Married	7	Islam	Primary	Housewife	More than 5 km
P11	29	Married	4	Christian	Primary	Local trader	More than 5 km
P12	30	Married	6	Islam	Primary	Housewife	More than 5 km

Abbreviation: CCT, conditional cash transfer.

### Data Collection

We conducted semi-structured interviews at the convenient time indicated by respondents. The interview guide (Supplementary file 1) was developed from available literature on the subject and a similar study on CCT conducted on maternal and newborn health in Nepal,^12^ given that similar published studies in Nigeria were not available at the time of this research. The questions varied from respondents’ decision to utilize the programme and what factors encouraged or discouraged their decisions. The guide was pre-tested and slightly adapted with each interview to improve flow of the interview and generate more information as themes evolved. Open-ended questions were used in the topic guide to allow themes to be developed and increase flexibility in response by participants.

We endeavoured to maintain participant confidentiality throughout the interview process. The importance of the study was explained to each participant with information sheets provided for literate participants and verbally explained to non-literate participants. The information sheets indicated study results would be anonymised as well as any direct quotes from participants. We ensured information sheets were well understood and consent sheets signed prior to the interview process with each participant. Informed consent was obtained from participants for audio recording of the interviews and consent forms signed. Interviews were all conducted in English except for two participants who only understood the Hausa language, which the primary researcher understood and spoke fluently.

Participants’ consent to audio recording was obtained prior to each interview, however, some declined and field notes were taken in these situations. Eight audio recordings and 12 field notes were obtained in total. Field notes also contributed to recording non-verbal data to facilitate the researcher’s memories of behaviours that might have been missed during recording.

Some of the participants were interviewed at the Byazhin PHC. However, these were done behind the facility to prevent service interruptions and distractions from the PHC health staff. The beneficiaries were interviewed first as they had been identified and contacted first from the SURE-P MCH database. Each interview lasted from thirty-five minutes to an hour and a half. Total interview duration for the study lasted about eight days. Repeat interviews were only conducted in one case where the participant was not providing adequate responses.

### Data Analysis

Data from the field notes and audio recordings were transcribed by the researchers and analysed daily after interviews by repeatedly listening to interview recordings, re-reading transcripts, and making preliminary observations. Inductive and deductive thematic analytic approaches were used.^26^ Data were analysed based on the following dimensions: experiences, facilitating factors and barriers to uptake of CCT services. Several overlapping dimensions arose during analysis. An MS-Word document was used to create visual aid and organizing participants quotes from the transcripts into themes.

## Results

Findings and responses have been disaggregated into five themes below (ie, programme perceptions, socio-cultural influences, physical access, programme administration, and health system factors). Some of the themes observed however overlap with one another and reasons provided by participants are due to multiple influences.

### Programme Perception

The perception of the initiative (including possible benefits) was a major determinant of response to the programme. Perceived programme objectives did not need to align with the true programme objectives to promote uptake. However, negative perception of government was an important deterrent.

Majority of the beneficiaries viewed the idea of the CCT programme positively and deemed it a necessary intervention by the government for the masses. However, most respondents did not fully comprehend the reasons behind CCT programme itself, eg, expected health gains and improving health-seeking behaviour even beyond the project.

Some of the beneficiaries stated the cash incentive was the main reason for utilizing the maternal and health services at the health facility as they felt excited by the idea of collecting cash for delivery at the PHC. Many perceived the programme as a substitute for unemployment among women.

*“When I first heard about it I said it was good of the government to finally remember us in the villages because like me now, I don’t work…the programme is a good one if they can continue it because it will show they are interested in the poor people and want to help us...”* [P1].

*“One of the CHEWs came to my house and told me the government will pay me to come for antenatal so I asked my neighbours and they said it is true… (name withheld) refused to go but I needed to collect the money, I have nothing else to do”* [P4].

Many respondents regarded the cash incentive as insufficient motivation with some indicating this as a deterring factor against continuing with the programme.

*“I heard they were giving money if you come to deliver here, so I came... I don’t know if I will come again (if the programme stops) …maybe, they (midwives) said its good for us but I don’t know...”* [P1].

*“…of course it is why I decided to come, I don’t work and my husband works very hard to feed us, anything extra will at least help me buy something for the house, but it’s not much you understand, they should give something more, you can’t really buy more than one or two things...”* [P3].

Most beneficiaries reported using the cash incentive to buy consumption goods for their family such as food ingredients, clothes, medicines for their other children, soap.

*“…I bought soap and some fish with the remaining money, my husband was happy with me...because I don’t work, he is always the one buying things for the house...”* [P3].

On the other hand, some non-beneficiaries cited a lack of trust in the government and disbelief the programme was real. One participant (non-beneficiary) had no reason for not registering for the CCT. She felt no need of going to the hospital as her previous children had all been born at home without complications.

*“(laughing...) why should I believe them, someone will just come and tell you they will give you money just go to the hospital, even if it is true, you will suffer and go today, tomorrow they will say sorry, the money has finished, no money for you…”* [P10].

*“Is it now after my five children that they will say come and collect N5000, when my last born was sick and I was borrowing money no government gave me money, my husband took him to (traditional herbalist), I can manage. They should pay for my five children then”* [P7].

### Socio-Cultural Influences

Positive spousal and community support for the CCT programme was a significant factor that further persuaded beneficiaries to register. All participants indicated strong husband influence in the decision to register or not register for the scheme. Two of the non-beneficiaries cited spousal disapproval as a reason for not registering for the scheme.

*“My husband doesn’t like the place (hospital), anyway he doesn’t even like me to go to my friends’ place, he said he will continue to pay for (Traditional Birth Attendant) to come home, after all, this one (points to child) was born without any problem at home”* [P10].

Local community and religious leaders who were aware of the benefits of the programme encouraged the women to register for the programme.

*“… so when I told my husband about it, he asked our pastor who said he has heard about it and his wife had also gone and registered, he said he will also tell other women…so we went with my husband*…” [P4].

Cultural practices (eg, home delivery by traditional birth attendants) influenced utilization of services, irrespective of incentives. One non-beneficiary regarded utilizing available health services as a last resort in the face of complications.

*“(proudly) All of us (female family members) give birth in our houses, even if I go for the ANC, I will still deliver at home because we don’t go to hospital to deliver…yes if there is problem I will go (to the PHC) but we don’t have problems...”* [P12].

### Programme Administration

Irregularity of cash transfers as well as unclear payment mechanism seemed to be a major concern. Beneficiaries were not aware of how or told when these payments would occur, they received phone calls from the health workers informing them of arranged payment dates.

*“They just call you on your phone to come…we give our phone numbers the first time we go there (for registration)”* [P1].

Contrary to the written program policy of four tranches staggered over pregnancy period, post-delivery and post-natal visits, payment was done in two tranches of unpredictable amounts totalling N5000.

*“The first time they called me to the facility (between ANC visits) and I was given N1300, the second time they gave me N3,700 (after delivery) ...”* [P2].

### Physical Access

A recurring theme cited as a reason for non-uptake of the CCT services among the non-beneficiaries was the distance from their homes.

*“If it wasn’t far, I would have gone but who will stay with the children when I go…nobody and I will go early in the morning, who will give them food then? They should bring another one near us in... (this village) and I will go, at least the children will be with me there”* [P7].

All the beneficiaries pointed out that they used part of the cash incentive for transport to the health facilities, though this was in the later part of their pregnancies as cash administration was paid only twice in between visits for ANC. Transport payments varied from N80 ($0.40) for the beneficiary living closest to the PHC to N500 ($2.50) for others on a public transport bike.

*“The only thing is the money I pay to the hospital you know, if not for that, we are happy with what they give us but by the time you remove money to pay for going, it is not much again*…” [P3].

*“I pay for transport to come here and go, all the time with my money, when they gave us (the cash), I paid from it then small (not much) left that I bought food for the house.*..” [P4].

Two of the non-beneficiaries complained about the cost of transport to the health facility as a deterring factor.

*“I live a long way from here..., I cannot trek to the hospital and I have to pay machine-man (bike) to carry me here, how much will the money remain (from N5000)….after I pay them to carry me and take me back? Every time I have to go, I will spend a long time on the road going…n*o” [P6].

*“…by the time I pay for transport, please how will I buy food for the house for one month?”* [P8].

### Health System Factors

Free maternal health services are provided at the PHCs under the SURE-P MCH scheme (free basic ANC services including drugs and delivery). This may have been an underlying factor encouraging uptake of the CCT. There are four midwives at each PHC facility employed by the SURE-P MCH program to provide maternal health services for the target community. Community health extension workers are also employed at the PHC but mainly conduct outreach services within the communities. Both beneficiaries and some non-beneficiaries who were aware of services provided at the facility complained of long waiting times for ANC services. There were also concerns about the quality of care received at the facilities and fears of poor treatment by the health workers.

*“There are too many women, even when I come very early in the morning, we have to join a long line (queue), it would be better if the place was bigger and they have more midwives to attend to us. The whole day will be spent waiting and waiting”* [P5].

*“If you get a wicked one (midwife) to deliver you, she will just be shouting at you for no reason, many women will tell you, and you can’t do anything because ...I will prefer to give birth at home at least I have my privacy and nobody will be shouting at me”* [P11].

## Discussion

Our study revealed that, although beneficiaries of the programme were influenced by the cash transfers, cash is regarded as an insufficient incentive for uptake of CCT by the majority of non-beneficiaries in Nigeria. Availability of free drugs, proximity to a health facility, spousal and community support are important factors that affect uptake. We also found barriers to uptake of CCT services in Nigeria to be similar to general barriers to demand for maternal health services. These findings are in consonance with similar studies in other LMICs. For example, the study of Vellakkal et al in India revealed that health workers’ support was a better determinant of service utilization than cash incentives. Further, as with our study, high opportunity costs, as well as scepticism, were found to be major deterrents to service utilization in spite of the CCT.^27^ However, our study further presents spousal and community influences as very important contextual determinants of service utilization in parts of Nigeria.

Assumptions that CCT is expected to affect changes in health behaviour is underpinned by some theoretical considerations.^28^ A typical model of provider choice demonstrates that when an individual is confronted with health and non-health choices, it is expected that the individual will make rational decisions to maximise health.^29^ However, behavioural economics has recently indicated limitations in human rationality due to a variety of factors.^30^ There are limits to consistency of the economic rationale of peoples’ behaviour indicated by deeply ingrained socio-cultural and health beliefs, physical and political economic structures.^29^

The decision of target beneficiaries regarding utilization of the CCT programme is affected by the complex interaction of multiple influences. For example, a participant who regards the health facility as too far to visit for maternal health services may also have misgivings regarding the intentions of the government programme. Private costs to the households well as opportunity costs are important influencers. If households deem these costs too high or higher than the provided incentives, it will serve as a deterrent from the uptake of the programme. Our findings in this regard are further supported by Powell-Jackson et al, and De Janvry and Sadoulet who posit that various contextual influences on demand for services must be put in consideration when implementing CCT programmes.^4,31^ The cash incentive consequently competes with multiple dynamics in form of sociocultural barriers, beliefs and human resource factors. This finding corroborates Adato and colleagues’ qualitative research on CCT programmes in four countries (Nicaragua, Mexico, El Salvador, and Turkey) which found beliefs on traditional and modern medical practices, gender relations, sociocultural norms and poverty experience to be important influences that compete with the cash incentives provided.^32^ Thus, cash incentives may not be as powerful a tool in predicting behaviour such as uptake of services contrary to widely held assumption. However, contextual evidence is required to understand variance in uptake determinants in diverse implementation environments.

Our findings reveal a similarity between barriers to CCT uptake in our Nigerian study context and general barriers to demand for maternal health services which include:

*Decision to seek care:* Distance and inability of the cash incentives to offset transport costs were among reasons cited by non-beneficiaries for deciding not to take advantage of the CCT programme. Thus, proximity to the health facility is an important finding which affects the women’s decision to utilize CCT services. In developing countries like Nigeria, this is especially a major factor where the density of modern facilities is low,^33^ and other nearby native sources of health services exist in the form of TBAs.^34^ Women might thus be inclined to use closer alternatives to formal health services. There is evidence in Nigeria and across the developing world that the distance that patients travel to access health services is a major deterrent in the utilisation of health services.^22,35-38^ This will likely affect the decision-making process even with the cash incentive as women who live far away would regard the opportunity costs of time spent travelling to the facility and time spent away from family, travel costs etc, as higher than the provided incentive.

Spousal involvement in the decision-making process for utilizing CCT services brings to light the need for involving males on a program’s intended objectives and benefits. Nigeria is a patriarchal society especially in the Northern regions of the country. This male dominance makes them primary decision makers regarding their spousal healthcare and health-seeking behaviour. Evidence points to the significant effect of husband’s role on decision making about the use of maternal health services.

The United Nations Population Fund (UNFPA) in 1997 explained the proactive roles men would play in maternal health services. The Mexico *Opportunidades* programme has involved males in its health education programme and this may have contributed to the programme’s observed impact.^32^

*Physical Accessibility:* Availability of health services differs from accessibility; the former refers to its physical existence while the latter is concerned with the degree to which healthcare can actually be attained when needed.^39^ If a community is located in a hard to reach region with poor transportation networks, a PHC located even a few kilometres away will serve as a major deterrent from accessing provided health services in spite of monetary incentives. Physical accessibility has been shown to significantly hinder utilization of health services in Nigeria, however one study showed that while Northern Nigeria has services located closer to population than Southern Nigeria, this does not reflect in health outcomes as the latter has significantly better population health indices.^38^ Consequently, other factors such as service availability and sociocultural/ economic factors may play more significant roles. Thus, the CCT programme may provide sufficient incentives if these other barriers are also addressed.

*Service Availability:* This is concerned with the actual provision of required health services at the facility. The availability of adequate number and availability of health workforce is an important consideration for optimum healthcare service delivery. This important consideration needs to be weighed before a country decides to roll out a CCT programme. The problem of health human resource availability at the PHCs have been attempted to be overcome by the SURE-P MCH CCT programme by employing additional female health workers in the form of midwives to provide additional maternal health services.^18^ However, it should be noted that PHCs are located in rural areas and midwives posted to these communities might have little in the way of motivation to provide adequate and quality health services to their patients.^40^ An unsatisfied and unmotivated health worker might not provide the type of treatment that will encourage women to continue to utilise available maternal health services even in the face of monetary incentives as revealed in this study. Quality of care in maternal and child health is an aspect of service delivery that involves “*providing a minimum level of care to all pregnant women and their newborn babies and a higher level of care to those who need it.*”^41^

Basic healthcare services provided at the PHCs are of low quality as reported by some of the respondents despite a supply-side aspect of the SURE-P MCH programme. Most respondents decried the poor quality of healthcare services provided at the PHC and some non-beneficiaries cited this perception as a reason for not registering for the CCT services provided. Studies across low-income countries allude that patient perception of quality is critical in the utilization of healthcare services.^42-44^ Improving the quality of care will lead to better patient satisfaction and consequently improve health seeking behaviour and utilisation of government-provided healthcare services like the CCT programmes. Generating awareness and improving commitment to quality of care among health workers is thus essential. However, this requires a strong leadership and commitment from the involved health workers.

Several facilitating factors encouraging uptake of the programme according to participants include approval of the policy. The indication was that it was the responsibility of the government to help the less privileged of the society through provision of the cash directly to the women. This might, however, lead to feelings of entitlement of the cash incentive which could hamper sustainability of positive health behaviour when the incentive is no longer available. It is therefore essential to provide knowledge on the far-reaching health gains associated with uptake of health services beyond the cash incentive associated with the programme.

Another factor cited by the beneficiaries as encouraging was the provision of free maternity care at the health facility in form of ANC drugs, contraceptive care post-delivery and free skilled attendance for uncomplicated deliveries. This further serves as an incentive to encourage women use available maternal services and register for the CCT as well. It signifies overcoming yet another obstacle to accessing healthcare, namely user fees which have been cited as a significant barrier to maternal health in many developing countries.^45^ Community and spousal awareness of this additional incentive also contribute to encouraging their pregnant women to utilize CCT services since there is no additional demand for payment of healthcare services.

Experiences of the beneficiaries with the programme point to problems with programme communication as they had a poor understanding of the aims of the CCT programme aside from receiving its immediate cash benefits and as such might revert to their original behaviours after the programme ends. These discoveries are significant in the Nigerian context where there is a general sense of government distrust as it will send even more negative signals and could further corrode public trust in the government’s future health programmes^20^ if the programme ends unexpectedly.

Beneficiaries’ accounts of the mechanism of cash payment varied from the stated policy, which stated that payment upon meeting conditions was to be made in four tranches (see Table 1 above). Most women reported that cash payments carried out by the health workers were provided in only two tranches of varying amounts and at unspecified times due to unknown reasons. The health workers placed calls to the beneficiaries to visit the facility for payments when the cash was available. This indicates there is existing uncertainty about the programme since the intent of the incentive is to overcome some of the financial barriers to accessing health service like transport costs. Also, women who register and are located in areas with poor mobile networks (a regular occurrence in the rural areas), might not be easily be reached to visit the PHC at these unscheduled times resulting in missed payments. This mode of cash also served as a disincentive as it meant women would have to pay for transport to the health facilities themselves before the cash incentive was paid.

Beneficiaries mainly reported using the cash provided to cater for immediate household needs. This finding is supported by a study by Cecchini and Madariaga who noted that there was associated increase in household consumption with the additional income provided by CCT.^46^ However, most studies on poverty reduction through CCT programmes do not indicate its effect in the long term. The finding highlights the difficulties linked with the capability of short-term directed interventions to lessen poverty in a sustainable way.

Non-beneficiaries who cited ignorance of the programme despite accessing the basic PHC services points to ongoing issues with communication/advocacy about the programme. Missed opportunities arising as a result of this through non-involvement of potential beneficiaries can be avoided by exploring other innovative ways of ensuring widespread dissemination of the programme within serving communities.

Our study has highlighted the importance of ensuring that quality health services (which communities are aware of and which are acceptable to them) as supply-side factors are put in place before implementing conditional cash programmes. Also, in a patriarchal society such as Northern Nigeria, it is critical to involve men when planning maternal health programmes as they play a significant role in the decision-making process to uptake the provided services. Health education is also required in rural communities with emphasis placed on the campaign of the goals of CCT to ensure they understand the need to utilize maternal health services even without the presence of cash incentives. Pre-planning estimated values of cash incentive should especially focus on transport costs borne by potential beneficiaries who live furthest away from the health facilities and in hard to reach areas. This will ensure that part of programme objectives of reducing financial barrier to accessing maternal healthcare with the use of the incentive is achieved.

We recommend that future studies should explore the results of this research in assessing the programme implementers’ perceptions in addition to target beneficiaries. This will assist in identifying bottlenecks associated with programme implementation and give a more rounded picture of factors that could affect uptake of CCT programmes. Further, for quantitative studies, measuring the impact of factors identified in this study on programme outcome in various regions of Nigeria will provide further evidence for effective programme implementation nationwide.

## Limitations

Our use of semi-structured interviews may have potentially restricted responses of the participants thus reflecting mostly superficial ‘official accounts’ rather than what they actually think. However, the use of semi-structured tools was important here, serving mostly as a guide to stimulate discussion. This method of open-ended discussions in a relaxed setting most likely stimulated participants to be more open about their real feelings. While focus group discussions would have been desirable to contribute to the data by generating new thinking about the topic by stimulating shy participants, semi-structured interviews also give participants the freedom to express themselves without pressure to agree with bolder participants.

The nature of qualitative studies which translates to a small number of study participants presents limitations in the external validity of the sample size, thus the study findings are not designed to be representative of the whole country. However, given the sociocultural similarities of the study population with the general population of North Central Nigeria, we view that findings in this study can effectively inform programme planning in the region.

## Conclusion

This study highlights the different factors affecting uptake of services and provides insight into the most important aspects of experiences of the CCT beneficiaries with the programme. A number of important lessons for policy emerge from the findings of this study that will be useful for future implementation of programmes. Contrary to widely held assumptions, this study provides evidence that cash incentives in CCT may not be as powerful a tool in predicting behaviour and in the uptake of services in Nigeria, particularly when they are regarded as insufficient. While different contexts call for different approaches, it is essential that maternal health services are available, accessible and of acceptable quality to target recipients for CCT programmes to reach their full implementation potential. Adequate mechanisms to widely disseminate information about a programme is required to avoid missing potential beneficiaries.

This study contributes to the global debates on alternative pathways to Universal Health Coverage by providing contextual considerations for strengthening demand-side strategies. Lessons learned could be applied to inform other related health financing strategies.

## Acknowledgements

Mylene Lagarde of the London School of Hygiene and Tropical Medicine, London, UK and Shamsuddeen Saad provided support and guidance. We also acknowledge the NPHCDA and SURE-P MCH staff for providing access to the contact details of all beneficiaries in the CCT scheme.

## Ethical issues

Informed consent was obtained from participants for audio recording of the interviews and consent forms signed. Interviews were all conducted in English except for two participants who only understood Hausa language, which the principal researcher understood and spoke fluently. The list of participants’ audio recording and transcripts are stored on the principal researcher’s personal computer in a password protected non-labelled file. Only the primary researcher has access to the study data. Formal ethical approval was obtained from the National Health Research Ethics Committee of Nigeria (NHREC) and London School of Hygiene and Tropical Medicine Ethics Board prior to commencing data collection for the study.

## Competing interests

Authors declare that they have no competing interests.

## Authors’ contributions

FBA designed the study and collected. FBA and EAE analyzed the data. All authors wrote the paper.

## Authors’ affiliations

^1 ^Department of Primary Healthcare Systems Development, National Primary Health Care Development Agency, Abuja, Nigeria. ^2^Presidential Initiative for the North East (PINE), Abuja, Nigeria. ^3^Department of Planning, Research and Statistics, National Primary Health Care Development Agency, Abuja, Nigeria. ^4^Centre for Health Policy, Wits School of Public Health, University of the Witwatersrand, Johannesburg, South Africa. ^5^Department of Global Health and Development, London School of Hygiene and Tropical Medicine, London, UK.

## Supplementary files

Supplementary file 1. Topic Guide for Factors Affecting Conditional Cash Transfer Uptake in North Central Nigeria.

## Key messages

**Implications for policy makers**

- This study will contribute to guiding policymakers in improving existing conditional cash transfer (CCT) programmes in Nigeria as well as inform more effective programming for planned CCT expansion projects.
- Policy-makers may be informed to include other demand-side factors into the CCT programme theory so as to eliminate real-world barriers to utilization of services by ensuring that maternal health services are available, accessible and of acceptable quality to target recipients.
- The importance of contextual realities is brought to fore in this study such that future programmes could be adapted to their implementation settings as against relying on generic programme models and theories.

**Implications for the public**

The findings in this study is an expression of real-world challenges of users of maternal health services in Nigeria. The findings reflect lived experiences of clients and will potentially inform programme redesign to suit user needs and priorities, thus acceptability and access to maternal health services.
