# Supplementary material for: Conditional Cash Transfers for Maternal Health Interventions: Factors Influencing Uptake in North-Central Nigeria
Source: Int J Health Policy Manag. 2018 Jun 25;7(10):934–42. doi: 10.15171/ijhpm.2018.56 (PMC6186474; doi:10.15171/ijhpm.2018.56)
Supplement: Supplementary file 1 — Topic Guide for Factors Affecting Conditional Cash Transfer Uptake in North Central Nigeria. [file ijhpm-7-934-s001.pdf]

Supplementary file 1. Topic Guide for Factors Affecting Conditional Cash Transfer Uptake in North Central Nigeria

A. **General experience**

1. How did you hear about the scheme?
2. Had you planned to deliver in the health facility before you heard about the scheme? If no, why did you change your mind?
3. Is the health facility far from your house? (how far is it and how do you get to the facility?)

B. **Factors affecting uptake of the scheme**

4. How did you make your decision to register (or not) for the programme?
5. What do you think of the cash incentive provided?
6. How did your immediate family, relatives, friends or other community members react to your decision? (Was there any support or disapproval for your decision)
7. How did this affect your decision to register?
8. What is your religion? (if any)
9. Is there any aspect of your religion that would encourage or discourage you from registering for the scheme? In what way?
10. What do the religious leaders in your community think about the scheme?

C. **Experiences with CCT service provision**

11. What do you know of the Conditional cash transfer programme? (what have you heard?)
12. What do you think of the programme?

13. What problems have you had with accessing the services of the programme?
14. What do you think is the most encouraging aspect of the programme?
15. How useful is the incentive provided to you? (how does it help you/your family?
16. Is the cash provided enough to cover transport to and from the facility?
17. What have you heard people discussing about the programme?
18. How do you think the programme could be improved? (what changes could be made that would have made you register –for non-beneficiaries)
19. Is there anything else you would like to add?
